# Lactate: The Fallacy of Oversimplification

**DOI:** 10.3390/biomedicines11123192

**Published:** 2023-12-01

**Authors:** Jiri Müller, Jaroslav Radej, Jan Horak, Thomas Karvunidis, Lenka Valesova, Miroslav Kriz, Martin Matejovic

**Affiliations:** Department of Internal Medicine, Faculty of Medicine in Pilsen, Charles University, Teaching Hospital Pilsen, Alej Svobody 80, 32300 Pilsen, Czech Republicvalesoval@fnplzen.cz (L.V.); matejovic@fnplzen.cz (M.M.)

**Keywords:** lactate, lactic acidosis, hyperlactatemia, shock, lactate metabolism, hypoxia, sepsis, septic shock

## Abstract

Almost a quarter of a millennium after the discovery of an acidic substance in sour milk by Swedish chemist Carl Wilhelm Scheele and more than 100 years after the demonstration of a tight connection between this lactic acid and tissue hypoxia in shock, we are still surrounded by false beliefs and misunderstandings regarding this fascinating molecule. Common perceptions of lactate, the conjugate base of lactic acid, as a plain waste product of anaerobic metabolism and a marker of cellular distress could not be further from the truth. Lactate is formed and utilized continuously by our cells, even under fully aerobic conditions, in large quantities, and although marked hyperlactatemia is always a red flag in our patients, not all these conditions are life-threatening and vice versa—not all critically ill patients have hyperlactatemia. Lactate also does not promote acidosis by itself; it is not toxic, nor is it a metabolic renegade. On the contrary, it has many beneficial properties, and an interpretation of hyperlactatemia might be trickier than we tend to think. The aim of this article is to debunk some of the deeply rooted myths regarding this fascinating molecule.

## 1. Introduction

Lactate is a multipurpose molecule produced during metabolism (both aerobic and anaerobic) in large quantities. Before we attempt to unravel some misconceptions, we must briefly summarize lactate metabolism in our cells. In the cytoplasm, glucose is converted into two pyruvate molecules and two redox equivalents (nicotin adenine dinucleotide—NADH). This process, known as glycolysis, does not require any oxygen regardless of its availability and provides two molecules of adenosine triphosphate (ATP). Under aerobic conditions, pyruvate and NADH (via glycerol–phosphate shuttle and/or malate–aspartate shuttle) are transported to the mitochondrial matrix. Pyruvate undergoes oxidative decarboxylation to form acetyl coenzyme A. Three enzymes that catalyze this reaction are integrated in the pyruvate–dehydrogenase complex. Its first subunit (pyruvate–dehydrogenase) is a multimeric protein with thiamine pyrophosphate as a cofactor. Thus, severe thiamine deficiency can lead to a cessation of this enzymatic process, and some evidence suggests that, apart from patients who are nutritionally incompetent, long-term furosemide treatment (common among the ICU population) may contribute to this condition due to significant urinary losses [1]. Acetyl coenzyme A enters the tricarboxylic acid cycle (also known as the Krebs cycle). All of these factors result in a total of 12 molecules of redox equivalents (10 NADH and 2 flavin adenine dinucleotides—FADH_2_), which together with 2 molecules of ATP from cytosolic glycolysis generate approximately 36 molecules of ATP in the electron-transport chain and subsequent adenosine triphosphate synthase activity. However, under anaerobic conditions, oxidative phosphorylation and the Krebs cycle slow. It is widely taught that, to regenerate an adequate amount of NAD^+^, pyruvate is converted via lactate–dehydrogenase into lactate, restoring normal redox potential (NADH to NAD^+^ ratio) and allowing for continuous (although poorly efficient) ATP production in glycolysis stimulated via phosphofructokinase by low ATP concentrations. Approximately one-half of this so called metabolic waste then enters the Cori cycle and shuttles to the liver, where hepatocytes reutilize lactate to form glucose again. The second half is removed by oxidation by different tissues, and only a small fraction is secreted to the urine. Simplistically, in the late 1980s, Cohen and Woods proposed that hyperlactatemia may arise from either insufficient oxygen delivery (type A hyperlactatemia) or from other causes not related to tissue hypoxia (type B hyperlactatemia) [2]. We incline toward a slightly different classification based on the mechanism of lactate elevation. Basically, excess lactate can accumulate under three circumstances: (a) increased pyruvate production (i.e., non-specific glycolysis stimulation by respiratory alkalosis, catecholamines, beta-agonists, etc.; malignancy-associated metabolic disturbances in the so-called Warburg effect; or other causes of the change in the redox cell potential of the cell, such as alcohol or diabetic ketoacidosis, as explained in following text); (b) decreased pyruvate utilization (i.e., hypoxia-related hyperlactatemia, the “type A” hyperlactatemia caused mainly by an insufficient load of oxygen in the blood or insufficient perfusion; pyruvate–dehydrogenase dysfunction typical of thiamine deficiency; or mitochondrial dysfunction, either inherited or acquired); and (c) decreased lactate clearance (predominantly in severe hepatic failure). Common causes are summarized (in a very simple way) in Table 1, but overlap between single causes surely exists. For example, animal experiments have shown that lactic acid infusion at rates similar to the rate of shock-associated lactate overproduction leads to non-significant acidosis and eventually normolactatemia, which is promoted by an increase in hepatic utilization of lactate. Therefore, shock-associated hyperlactatemia is probably multifactorial, and diminished hepatic and/or renal clearance plays a role in the pathogenesis.

Lactate is widely perceived to be a waste product and a danger-signaling molecule. Moreover, since lactic acid is relatively strong, it promotes lactic acidosis with a concurrent drop in pH. This deceptively easy concept is in many ways misleading, as we attempt to show in the following lines.

## 2. Main Text

**Myth 1: Hyperlactatemia in (septic) shock is an undisputed sign of hypoperfusion and tissue hypoxia.**

Shock is defined as a state of acute tissue hypoperfusion, leading to inadequate coverage of metabolic demands. Hence, lactate is valued as a sensitive marker of cell stress, and even mild hyperlactatemia is associated with increased mortality in critically ill patients [3]. The central position of lactate is further solidified in the Sepsis-3 definition of septic shock, a subset of sepsis that can be identified by the vasopressor requirement to maintain a mean arterial pressure of 65 mm Hg or greater and a serum lactate level greater than 2 mmol/L in the absence of hypovolemia. Measurement of the lactate level is currently recommended as part of the initial evaluation of sepsis. It has been suggested that lactate can be used to recognize patients with “occult” shock [4].

Although we acknowledge this connection between hyperlactatemia and septic shock, there is a scarcity of data regarding the connection between oxygen debt and hyperlactatemia, originally proposed by Weil [5]. Several studies have shown that partial oxygen pressure in muscles and other tissues is normal or even mildly elevated in patients with septic shock [6,7,8]. Moreover, some authors have suggested that one of the key producers of lactate in sepsis and septic shock are the lungs [9,10], and in contrast, splanchnic lactate release is minimal, even in profound hyperlactatemia [11]. Although the exact source of lactate generation during sepsis remains controversial, it is safe to say that the lungs are certainly the most oxygenated organ in the human body. Hypoxia, therefore, seems unlikely to be the main cause of hyperlactatemia, although a fraction of lactate elevation might contribute to microcirculatory shunting [12]. Thus, what exactly is the main cause of hyperlactatemia in septic shock? A growing body of evidence regarding sepsis-associated mitochondrial dysfunction has arisen. We now know that several mechanisms, such as reactive oxygen and nitrogen species [13,14], inflammation-induced changes in gene transcription, or metabolic changes, such as free-fatty acid accumulation and lipotoxicity caused by dysfunction of beta oxidation in lipopolysaccharide (LPS)-induced reduction in the PPAR alpha signaling cascade [15], participate in this phenomenon. Simultaneously, the activity of pyruvate–dehydrogenase, an enzyme complex responsible for the conversion of pyruvate into acetyl coenzyme A, is impaired in later stages of septic shock, possibly due to cytokine-induced up-regulation of pyruvate dehydrogenase kinases, which decrease the flux of the pyruvate dehydrogenase complex [16,17]. Proving this point, an infusion of dichloroacetate, an activator of pyruvate dehydrogenase, has been shown to reduce hyperlactatemia in septic shock [18]. Mitochondrial dysfunction explains the hyperlactatemia in late stages of shock, but as we have already mentioned, lactate is a potential sign of occult shock, which is a clinical scenario in which we typically do not come across profound mitochondrial dysfunction. Why is lactate elevated then? To answer this question, one must look a bit more deeply into lactate’s origin. Lactate is a crucial intermediate of not only anaerobic but also aerobic metabolism. During glycolysis, some lactate is generated, and the rate of this generation is dependent on: (a) the redox state of the cell or, better stated, the NADH-to-NAD+ ratio; and (b) lactate dehydrogenase’s substrate genesis, which is pyruvate, and therefore glycolytic flux. Glycolysis is internally inhibited by acetyl coenzyme A, citrate adenosine triphosphate, and H+ and is stimulated by adenosine monophosphate in an allosteric manner. Externally, the main stimulator is adrenaline via cyclic adenosine monophosphate. This adrenergic drive is probably the cause of hyperlactatemia in early phases of shock, when organism stress reactions and endogenous catecholamine release compensate for hypotension. In line with this theory, lactate elevation has been repeatedly observed after exogenous epinephrine administration [19,20]. Therefore, hyperlactatemia may be perceived mainly as a valuable marker of cellular stress response and an early warning sign of compensated shock, similar to sinus tachycardia. Indeed, in acutely ill patients presenting with deceptively reassuring vital signs, lactate measurements might be helpful in identifying a subset of patients at high risk for further deterioration.

**Myth 2: Hyperlactatemia is always a sign of a life-threatening condition.**

Metaphorically, lactate in shock is equal to cardiac troponin in myocardial infarction. On the one hand, it represents a valuable and sensitive window into the cell metabolism, but on the other hand, it is prone to error and misinterpretation since there are many causes of hyperlactatemia, and not all of them are shock or other life-threatening conditions. This fact unfortunately also applies in the opposite direction—not all critically ill septic patients have hyperlactatemia [21].

We already mentioned the influence redox state inside the cell determined by the NADH-to-NAD^+^ ratio, which reflects the proportion between lactate and pyruvate. The typical ratio of lactate to pyruvate is 10:1. Hyperlactatemia may represent either a disturbance of this ratio caused by lactate (and NADH) accumulation or the lactate elevation being coupled with pyruvate in the same proportion. NADH accumulation may occur not only in hypoxia and respiratory chain dysfunction but also in the case of metabolism of certain substances, such as ethanol or ketone bodies. This process leads to mild hyperlactatemia in active alcohol abuse and diabetic ketoacidosis. Likewise, drugs inhibiting the respiratory chain (i.e., linezolid, metformin, propofol) can cause NADH and lactate accumulation.

Glycolytic flux is adrenergic driven, but not only endogenous catecholamines mediate this action as a part of shock compensation or exogenous vasopressor administration. Pheochromocytoma, a catecholamine-secreting tumor, may cause severe hyperlactatemia [22], and the same applies to inhaled beta-2-mimetics in the treatment of bronchial asthma or chronic obstructive pulmonary disease [23]. Apart from adrenergic stimuli, a pH elevation also spins the wheels of glycolysis; therefore, respiratory alkalosis causes lactate elevation. This fact can be observed in panic attacks [24], as well as in the case of salicylate toxicity, with direct central stimulation of ventilation [25] combined with cell-metabolism disturbances.

Moreover, some authors have proposed that long-term beta-blocker therapy might decrease lactate concentrations in acute states. In a retrospective study conducted by Contenti et al. in patients presenting to the emergency department with sepsis and/or septic shock, blood lactate was significantly lower in the group previously treated with beta-blockers, and beta-blockade was (apart from survival) the only factor independently associated with normal lactatemia [26]. Thus, long-term beta-blockade might underestimate the severity of sepsis/septic shock triage, not only by modulating potential tachycardia but also by lowering lactate concentrations.

**Myth 3: Lactate is a cause of acidosis.**

Some textbook phrases are widely perceived as unquestionable truth. In otherwise respectable sources, we can read sentences such as “*Lactic acidosis is the most common cause of metabolic acidosis in hospitalized patients*” [27]. However, if we look a bit closer, this belief starts to decay. First, there is actually just a fraction of lactic acid systemically since lactic acid with pK_a_ (dissociation constant) of 3.86 is similar to “pyruvate acid” in being almost completely ionized at a normal pH, and no protons are generated in lactate genesis from pyruvate. In fact, paradoxically, during this reaction, two protons are consumed, which is the exact opposite of what one would expect. During glycolysis, a total of two protons in the form of NADH + H^+^ and two pyruvate molecules are generated. Those redox equivalents are utilized in the respiratory chain (under aerobic conditions) or in lactate formation (under anaerobic conditions). Therefore, although glycolysis itself is acidifying, subsequent metabolic steps lead to net zero proton formation. Thus, what is the cause of acidosis in hyperlactatemia associated with sepsis? The answer is simple—production of lactate by means of glycolysis is inevitably associated with the release of an equivalent number of protons from the hydrolysis of adenosine triphosphate generated during glycolysis [28]. Under standard aerobic circumstances, those protons are consumed by adenosine triphosphate synthase in oxidative phosphorylation. In the case of hypoxia or mitochondria dysfunction, this outcome is impossible, and the protons generated by adenosine triphosphate hydrolysis accumulate, causing a drop in pH. This fact explains why it is not uncommon to spot a patient with hyperlactatemia without acidosis. Hence, the term *lactate acidosis* is inaccurate, implying that lactate is acidifying. In reality, “lactate-associated” acidosis would be more suitable because lactate is not responsible for acidosis. In sepsis, it seems that this lactate-associated acidosis is compensated for primarily by the kidneys, with a decrease in strong anions widening a strong ion difference. Hence, the degree of acidemia heavily depends upon renal function [29]. Keeping this point in mind, there are still situations leading to lactic acidosis with lactic acid generation, as rightly pointed out by Qian [30], but locally and under “real” hypoxemic conditions, typically in a working skeletal muscle. However, lactate is produced in skeletal muscle mainly by type IIa and IIx muscle fibers, whereas type I muscle fibers can utilize this lactate in an oxidative manner, albeit in a much slower process.

**Myth 4: Lactate-associated acidosis is a cause of hyperkalemia.**

We all are familiar with the work of Burnell et al., who proved for the first time that acidosis leads to hyperkalemia, as confirmed in subsequent studies [31]. This internal potassium balance is usually explained simply by the law of electroneutrality—the buffering capacity of cells leads to an influx of hydrogen ions intracellularly, and to balance the charge difference, potassium exits the cells and accumulates. However, in the case of organic anions (such as ketone bodies or lactate), this process does not apply [32]. The reason why not is possibly robust inward flux of those anions inside the cell, leading to intracellular acidemia stimulating Na^+^/K^+^ ATPase with a net gain in cell potassium. This process may lead to normokalemia or even hypokalemia in a patient with lactic acidosis or diabetic ketoacidosis [33]. The hyperkalemia that we sometimes see in patients with DKA is more likely induced by insulinopenia (and low Na^+^/K^+^ ATPase turnaround) and hyperosmolarity with a subsequent “solute-drag” mechanism, rather than pH-related changes. A counter-mechanism to this process is the loss of potassium in the kidneys associated with ketone body excretion, which leads to hypokalemia rather than hyperkalemia in a typical patient with diabetic ketoacidosis. This outcome, combined with insulin administration in the treatment of ketoacidosis, may induce severe, life-threatening hypokalemia. All in all, clinicians treating patients with hyperlactatemia must pursue other explanations of potential hyperkalemia than only lactic acidosis.

**Myth 5: Lactate clearance can be used as a guide in resuscitation of patients with shock.**

First, let us discuss the term *lactate clearance*. Clearance is defined as the volume of plasma from which a substance is removed per unit of time. However, lactatemia is a result of continuous production, oxidation as a source of energy, and reutilization for gluconeogenesis. The kidneys, although they are alongside the liver, which is the organ responsible for the largest part of lactate removal, utilize lactate in their metabolism via renal cortex uptake, rather than simply excreting it in the urine [34]. Therefore, the term *clearance* when talking about lactatemia is again misleading; in the real world, it is impossible to determine whether the decline in lactatemia is due to “clearance” or a decrease in production, reutilization, or another metabolic process.

Current Surviving Sepsis Campaign guidelines suggest guiding resuscitation in sepsis or septic shock to decrease serum lactate, and there is some evidence to back up this suggestion [35,36]. The question is: what are we aiming at? Sure, hyperlactatemia is undoubtedly a marker of severity in a large sample of the population, but at an individual level, the complexity of this molecule makes it difficult or even impossible to interpret solely the dynamic changes of lactate levels with certainty. Lowering lactate is not a holy grail of resuscitation since it has no direct association with hemodynamic improvement, energetic failure reversion, or restoration of adequate perfusion. The goal of therapy is not to normalize lactate concentrations but to interrupt the pathological process causing lactate to increase.

**Myth 6: For lactate measurement we should use only arterial blood sample and point-of-care analyzers.**

Not really—both venous and arterial blood can be used and both seem to have a high level of agreement [37]. This fact is of course conditional upon a correct pre-analytical phase. The blood sample for lactate testing should be drawn first after tourniquet application, as lactate levels can be elevated, with prolonged blood flow restriction. If the sample is tested immediately on the blood gas analyzer, there is no need to put it on ice. In fact, at least in healthy subjects, lactate concentrations remain practically unchanged for 15 min at room temperature [38].

The lactate gap between laboratory and point-of-care methods may occur under specific circumstances. The reason for this outcome is a different way of detecting lactate—most bedside analyzers utilize a lactate–oxidase-based system, while laboratories utilize a lactate–dehydrogenase-based system. This fact has important consequences in alcohol poisoning. The ethylene glycol metabolite glycolate mimics the chemical structure of lactate, and portable analyzers using non-selective lactate–oxidase may report falsely high lactatemia. Therefore, this pseudo-lactic acidosis and lactate gap may assist in the diagnosis of ethylene glycol intoxication [39].

**Myth 7: Lactate is a waste product of cell metabolism.**

Lactate has been viewed as a waste product of hypoxia; however, in light of current knowledge, we must accept that lactate is an essential source of energy, plays a crucial role in cellular signaling, and possibly influences gene transcription in an epigenetics manner.

The heart utilizes lactate even at rest, but in hyperlactatemia, lactate may even exceed glucose as the main source of pyruvate and energy [40,41]; similarly, the brain covers up to one-quarter of energetic requirements with lactate [42]. Interestingly, sodium lactate infusion improves cardiac output in patients with acute heart failure [43]. Moreover, organ-to-organ and cell-to-cell shuttle of lactate via monocarboxylate transporters has been proposed as an efficient mechanism for energy redistribution between lactate-producing and lactate-consuming cells and tissues [44]. Apart from entering cells to serve as an energy source, lactate also represents an important signaling molecule. After binding to a G protein-coupled receptors localized mainly in the adipose tissue, kidneys, skeletal muscle, central nervous system, and cardiomyocytes, lactate mediates neuronal protection, inflammatory regulation, and lipolysis [45]. In addition to two well-known metabolic pathways (oxidation and gluconeogenesis), lactate can be converted into lactyl coenzyme A, and this form of coenzyme A may be involved in the lactylation of histones and non-histone proteins. In 2019, Zhang et al. demonstrated for the first time a new type of this epigenetic modification. Lactylated histones have been found in cells stimulated by hypoxia, interferon-gamma, and LPS, thereby implying direct gene expression regulation by lactate [46]. There is still much to determine regarding lactate function in the human body, but it is clear that lactate represents far more than only metabolic waste.

**Myth 8: Ringer-lactate, Isolyte, or Hartmann solutions shouldn’t be used in pre-existing hyperlactatemia.**

The discussion between normal saline and balanced crystalloids in fluid resuscitation seems to be over, uncovering yet another theme to argue about—various anions in intravenous fluids. Four main anions are currently present in balanced crystalloids: lactate (*Ringer-lactate*, *Hartmann*), acetate (*Plasmalyte*, *Isolyte*, *Ringerfundin*), gluconate (*Plasmalyte*, *Isolyte*), and malate (*Ringerfundin*). Historically, the administration of sodium lactate was feared in cases of pre-existing hyperlactatemia due to its hypothetically worsening lactic acidosis. This is simply not the case—sodium lactate is not an acid, and a strong ion difference after lactate anion metabolism via hepatocytes leads to physiologic pH values (unlike the “normal” saline with a strong ion difference equal to zero). Lactate levels might theoretically rise although probably only under experimental conditions while infusing large volumes [47]. The lactate concentration in *Ringer-Lactate* is around 30 mmol/L, that is, significantly lower than the daily production of lactate in our metabolism, which is roughly around 20 mmol/kg/day [48]. However, in case of fulminant hepatic or bioenergetic failure (i.e., in metformin-induced lactic acidosis), one might consider using balanced crystalloids without lactate. With that being said, there is probably not an “optimal” fluid in severe bioenergetic failure since, for example, *Plasmalyte* contains acetate, which might also accumulate. To date, no relevant data showing superiority of any solution are available.

**Myth 9: Lactic acidosis is always accompanied by (L-)lactate elevation.**

When we talk about “lactic acidosis,” we refer to a specific enantiomer—L-lactate. However, D-lactate can, under specific conditions, cause significant high-anion gap acidosis with clinical symptoms. The pitfall of this outcome is that the enzymatic laboratory methods for lactate measurement detect only L-isomer; therefore, lactic acidosis without (L-)lactate elevation may occur. Three main settings in which D-lactic acid accumulates are: (a) a patient with short bowel syndrome after consumption of large amounts of carbohydrates; (b) a patient exposed to a great load of propylene glycol; and (c) a patient with diabetic ketoacidosis.

Short bowel syndrome (SBS) is a state of malabsorption caused by substantial resection of the small intestine. Carbohydrates delivered to the colon, without being processed in the small intestine, are metabolized by bacteria into D-lactic acid, which is absorbed in large quantities. A co-factors for D-lactic acid production are intestinal dysbiosis with Gram-positive anaerobe overgrowth (i.e., lactobacilli) and lower intraluminal pH, which create ideal conditions for D-lactate-producing bacteria [49]. Therefore, patients with SBS have chronic asymptomatic low-grade D-lactic acidemia [50]. After carbohydrate loading, a peak in D-lactate concentrations occurs, and episodic neurologic abnormalities (confusion, slurred speech, ataxia, etc.) may manifest. That the degree of clinical symptoms does not correlate with D-lactic acid levels implies that there are also other toxins produced in the intestine, which are co-absorbed with D-lactate and are (at least partially) responsible for clinical symptoms [51]. Propylene glycol is a viscous fluid used as a solvent for a wide variety of medications. A high rate of infusion of these drugs may cause this diol to accumulate and cause an osmolal gap, followed by anion gap acidosis caused by D-lactic acid, which is a metabolic product of propylene glycol. There have been some case reports about significant acidemia caused by this phenomenon, mainly by high benzodiazepine infusion [52]. Lu et al. [53] reported high levels of D-lactic acid in patients with diabetic ketoacidosis, probably caused by methyl–glyoxal (metabolite of acetone and dihydroxy acetone phosphate) metabolism, but the extent to which D-lactic acid influences patients with ketoacidosis remains unknown.

## 3. Conclusions

This text should provide partial insight into lactate metabolism and overcome some prejudices connected with this multipurpose cell mediator. From a practical standpoint, taking away all of the pathophysiological glitter, it is crucial to determine whether hyperlactatemia is a marker of cellular stress or a simple by-product of metabolism with many beneficial properties. In the first case, we should ideally be able to distinguish the predominant oxygen delivery issue (i.e., profound anemia, shock states or mesenteric ischemia) from oxygen use impairment and/or altered lactate metabolism (i.e., intoxication, sepsis or liver injury) since the therapeutic approach will be different.

As simple as it sounds, we often find ourselves struggling with the correct interpretations at the bedside since the explanation is mostly not straightforward, and multiple factors must be considered. When evaluating lactate elevation, we follow five main statements, which are applicable to practically any patient, critically ill or not. (1) **Be careful**. Marked hyperlactatemia is always a red flag, and every patient with a significant lactate elevation deserves our maximum attention. Especially in the absence of a clear explanation, we must exclude hidden, but potentially life-threatening, conditions associated with an increase in lactate production (i.e., occult shock, intoxication, or on-going ischemia). Some medications might alter lactate concentrations, and normolactatemia does not necessarily imply that the patient is not in severe condition. (2) **Be respectful**. Lactate is a strong marker of cellular stress that might suggest emerging or actual critical illness. Elevated lactate levels are clearly associated with increased morbidity and mortality in acutely ill patients. (3) **Be doubtful**. A glycolytic increase in lactate concentration is found in many metabolic states, and certainly not all of them are life-threatening conditions. Moreover, although an increase in lactate levels during treatment of (septic) shock calls for re-evaluation, it does not necessarily indicate the need for more aggressive resuscitation. This principle also applies the other way around—not all critically ill patients have hyperlactatemia. (4) **Be mindful**. Lactate is formed and utilized continuously by our cells under fully aerobic conditions in large quantities. It does not promote acidosis per se, and it is not toxic or metabolic waste; on the contrary, it is an important bioenergetic substrate and multifunctional signaling molecule with anti inflammatory, antioxidant, and immunomodulatory properties. (5) **Be crafty (or obtain help)**. Knowledge is required for, but is not a guarantee of, success. Interpretation of hyperlactatemia might be difficult, and the degree of lactate elevation does not necessarily correlate with disease severity or prognosis. Hence, every deteriorating patient with hyperlactatemia should be reviewed by a clinician experienced in managing such patients.

## Figures and Tables

**Table 1:** Classification of hyperlactatemia.

**increased pyruvate production**	**non-specific glycolysis stimulation**	respiratory alkalosis, catecholamines (mainly epinephrine), inhaled beta-agonists, pheochromocytoma	
	**malignancy-associated metabolic disturbances (*Warburg effect*)**	leukemia, lymphoma, or, less often, solid malignancies	
	**other causes influencing the redox potential of the cell**	alcoholism, diabetic ketoacidosis	
**decreased pyruvate utilization**	**hypoxia-related hyperlactatemia (type A hyperlactatemia)**	crossing of an anaerobic threshold in skeletal muscle	intensive muscle activity, generalized convulsions, hypothermic shivering
		insufficient load of oxygen in blood	hypoxemic hypoxia, profound anemia, CO intoxication
		insufficient perfusion	shock, regional ischemia (i.e., mesenteric ischemia), cardiac arrest, microvascular shunting
	**pyruvate-dehydrogenase dysfunction**	thiamine deficiency (beriberi), inherited enzymatic dysfunction	
	**mitochondrial dysfunction**	drugs (propofol, linezolid, metformin, etc.), inherited disorders, cyanide intoxication, sepsis	
**altered clearance of lactate**	liver failure, renal failure		

## Data Availability

Not applicable.

## Abbreviations

**ATP**—adenosine triphosphate, **NADH**—nicotin adenine dinucleotide, **FADH_2_**—flavin adenine dinucleotide, **ICU**—intensive-care unit, **LPS**—lipopolysaccharide, **PPAR**—peroxisome proliferator-activated receptor, **pK_A_**—acid dissociation constant, **DKA**—diabetic ketoacidosis, **SBS**—short bowel syndrome

## References

[1] Seligmann H., Halkin H., Rauchfleisch S., Kaufmann N., Tal R., Motro M., Vered Z., Ezra D. (1991). Thiamine deficiency in patients with congestive heart failure receiving long-term furosemide therapy: A pilot study. Am. J. Med..

[2] Cohen R.D., Woods H.F. (1983). Lactic acidosis revisited. Diabetes.

[3] Rishu A.H., Khan R., Al-Dorzi H.M., Tamim H.M., Al-Qahtani S., Al-Ghamdi G., Arabi Y.M. (2013). Even mild hyperlactatemia is associated with increased mortality in critically ill patients. Crit. Care.

[4] Evans L., Rhodes A., Alhazzani W., Antonelli M., Coopersmith C.M., French C., Machado F.R., Mcintyre L., Ostermann M., Prescott H.C. (2021). Surviving sepsis campaign: International guidelines for management of sepsis and septic shock 2021. Crit. Care Med..

[5] Weil M.H., Afifi A.A. (1970). Experimental and clinical studies on lactate and pyruvate as indicators of the severity of acute circulatory failure (shock). Circulation.

[6] Boekstegers P., Weidenhöfer S., Kapsner T., Werdan K. (1994). Skeletal muscle partial pressure of oxygen in patients with sepsis. Crit. Care Med..

[7] Sair M., Etherington P.J., Peter Winlove C., Evans T.W. (2001). Tissue oxygenation and perfusion in patients with systemic sepsis. Crit. Care Med..

[8] Levy B., Gibot S., Franck P., Cravoisy A., Bollaert P.-E. (2005). Relation between muscle Na+K+ ATPase activity and raised lactate concentrations in septic shock: A prospective study. Lancet.

[9] Opdam H., Bellomo R. (1999). Oxygen Consumption and Lactate Release by the Lung after Cardiopulmonary Bypass and during Septic Shock. Crit. Care Resusc..

[10] Kellum J.A., Kramer D.J., Lee K., Mankad S., Bellomo R., Pinsky M.R. (1997). Release of lactate by the lung in acute lung injury. Chest.

[11] De Backer D., Creteur J., Silva E., Vincent J.L. (2001). The hepatosplanchnic area is not a common source of lactate in patients with severe sepsis. Crit. Care Med..

[12] Spronk P.E., Zandstra D.F., Ince C. (2004). Bench-to-bedside review: Sepsis is a disease of the microcirculation. Crit. Care.

[13] Singer M. (2017). Critical illness and flat batteries. Crit. Care.

[14] Singer M. (2007). Mitochondrial function in sepsis: Acute phase versus multiple organ failure. Crit. Care Med..

[15] Van Wyngene L., Vandewalle J., Libert C. (2018). Reprogramming of basic metabolic pathways in microbial sepsis: Therapeutic targets at last?. EMBO Mol. Med..

[16] Nuzzo E., Berg K.M., Andersen L.W., Balkema J., Montissol S., Cocchi M.N., Liu X., Donnino M.W. (2015). Pyruvate dehydrogenase activity is decreased in the peripheral blood mononuclear cells of patients with sepsis. A prospective observational trial. Ann. Am. Thorac. Soc..

[17] Alamdari N., Constantin-Teodosiu D., Murton A.J., Gardiner S.M., Bennett T., Layfield R., Greenhaff P.L. (2008). Temporal changes in the involvement of pyruvate dehydrogenase complex in muscle lactate accumulation during lipopolysaccharide infusion in rats. J. Physiol..

[18] Stacpoole P.W., Nagaraja N.V., Hutson A.D. (2003). Efficacy of dichloroacetate as a lactate-lowering drug. J. Clin. Pharmacol..

[19] Totaro R.J., Raper R.F. (1997). Epinephrine-induced lactic acidosis following cardiopulmonary bypass. Crit. Care Med..

[20] Xue J., Mannem S., Al Jandeel A., Ruvo A., Levi D. (2020). Epinephrine causes severe lactic acidosis in a patient with shellfish-induced anaphylaxis. Chest.

[21] Sauer C.M., Gómez J., Botella M.R., Ziehr D.R., Oldham W.M., Gavidia G., Rodríguez A., Elbers P., Girbes A., Bodi M. (2021). Understanding critically ill sepsis patients with normal serum lactate levels: Results from U.S. and European ICU cohorts. Sci. Rep..

[22] Manoj Kumar R.M., Narayanan N.K., Raghunath K.J., Rajagopalan S. (2019). Composite pheochromocytoma presenting as severe lactic acidosis and back pain: A case report. Indian J. Nephrol..

[23] Rodrigo G.J., Rodrigo C. (2005). Elevated plasma lactate level associated with high dose inhaled albuterol therapy in acute severe asthma. Emerg. Med. J..

[24] de Ridder S., Kuijpers P., Crijns H. (2010). Lactate: Panicking doctor or panicking patient?. BMJ Case Rep..

[25] Blohm E., Lai J., Neavyn M. (2017). Drug-induced hyperlactatemia. Clin. Toxicol..

[26] Contenti J., Occelli C., Corraze H., Lemoël F., Levraut J. (2015). Long-term β-blocker therapy decreases blood lactate concentration in severely septic patients. Crit. Care Med..

[27] Emmett M., Szerlip H., Sterns R.H., Forman J.P. (2023). Causes of Lactic Acidosis. UpToDate.

[28] Kraut J.A., Madias N.E. (2014). Lactic acidosis. N. Engl. J. Med..

[29] Gattinoni L., Vasques F., Camporota L., Meessen J., Romitti F., Pasticci I., Duscio E., Vassalli F., Forni L.G., Payen D. (2019). Understanding lactatemia in human sepsis. Potential impact for early management. Am. J. Respir. Crit. Care Med..

[30] Qian Q. (2018). Reply to Robergs et al. Physiology.

[31] Burnell J.M., Scribner B.H., Uyeno B.T., Villamil M.F. (1956). The effect in humans of extracellular pH change on the relationship between serum potassium concentration and intracellular potassium. J. Clin. Investig..

[32] Fulop M. (1979). Serum potassium in lactic acidosis and ketoacidosis. N. Engl. J. Med..

[33] Aronson P.S., Giebisch G. (2011). Effects of pH on potassium: New explanations for old observations: New explanations for old observations. J. Am. Soc. Nephrol..

[34] Bellomo R. (2002). Bench-to-bedside review: Lactate and the kidney. Crit. Care.

[35] Gu W.-J., Zhang Z., Bakker J. (2015). Early lactate clearance-guided therapy in patients with sepsis: A meta-analysis with trial sequential analysis of randomized controlled trials. Intensive Care Med..

[36] Vincent J.L., Quintairos e Silva A., Couto L., Taccone F.S. (2016). The value of blood lactate kinetics in critically ill patients: A systematic review. Crit. Care.

[37] Middleton P., Kelly A.-M., Brown J., Robertson M. (2006). Agreement between arterial and central venous values for pH, bicarbonate, base excess, and lactate. Emerg. Med. J..

[38] Jones A.E., Leonard M.M., Hernandez-Nino J., Kline J.A. (2007). Determination of the effect of in vitro time, temperature, and tourniquet use on whole blood venous point-of-care lactate concentrations. Acad. Emerg. Med..

[39] Sagar A.S., Jimenez C.A., Mckelvy B.J. (2018). Lactate gap as a tool in identifying ethylene glycol poisoning. BMJ Case Rep..

[40] Matejovic M., Radermacher P., Fontaine E. (2007). Lactate in shock: A high-octane fuel for the heart?. Intensive Care Med..

[41] Beadle R.M., Frenneaux M. (2010). Modification of myocardial substrate utilisation: A new therapeutic paradigm in cardiovascular disease. Heart.

[42] van Hall G. (2010). Lactate kinetics in human tissues at rest and during exercise: Tissue lactate kinetics. Acta Physiol..

[43] Nalos M., Leverve X., Huang S., Weisbrodt L., Parkin R., Seppelt I., Ting I., Mclean A.S. (2014). Half-molar sodium lactate infusion improves cardiac performance in acute heart failure: A pilot randomised controlled clinical trial. Crit. Care.

[44] Brooks G.A. (2018). The science and translation of lactate shuttle theory. Cell Metab..

[45] Li X., Yang Y., Zhang B., Lin X., Fu X., An Y., Zou Y., Wang J.-X., Wang Z., Yu T. (2022). Lactate metabolism in human health and disease. Signal Transduct. Target. Ther..

[46] Zhang D., Tang Z., Huang H., Zhou G., Cui C., Weng Y., Liu W., Kim S., Lee S., Perez-Neut M. (2019). Metabolic regulation of gene expression by histone lactylation. Nature.

[47] Boysen S.R., Dorval P. (2014). Effects of rapid intravenous 100% L-isomer lactated Ringer’s administration on plasma lactate concentrations in healthy dogs: In vivo effects of lactated Ringer’s in healthy dogs. J. Vet. Emerg. Crit. Care.

[48] Connor H., Woods H.F. (1982). Quantitative aspects of L(+)-lactate metabolism in human beings. Ciba Foundation Symposium 87—Metabolic Acidosis.

[49] Caldarini M.I., Pons S., D’Agostino D., Depaula J.A., Greco G., Negri G., Ascione A., Bustos D. (1996). Abnormal fecal flora in a patient with short bowel syndrome: Anin vitro study on effect of pH ond-lactic acid production. Dig. Dis. Sci..

[50] Bongaerts G., Tolboom J., Naber T., Bakkeren J., Severijnen R., Willems H. (1995). D-lactic acidemia and aciduria in pediatric and adult patients with short bowel syndrome. Clin. Chem..

[51] Uribarri J., Oh M.S., Carroll H.J. (1998). D-lactic acidosis: A review of clinical presentation, biochemical features, and pathophysiologic mechanisms. Medicine. Medicine.

[52] Tsao Y.-T., Tsai W.-C., Yang S.-P. (2008). A life-threatening double gap metabolic acidosis. Am. J. Emerg. Med..

[53] Lu J., Zello G.A., Randell E., Adeli K., Krahn J., Meng Q.H. (2011). Closing the anion gap: Contribution of d-lactate to diabetic ketoacidosis. Clin. Chim. Acta.

